# Hydrocéphalie de l’enfant: aspects clinique, paraclinique et thérapeutique dans quatre formations médicales de Lubumbashi

**DOI:** 10.11604/pamj.2022.43.114.27919

**Published:** 2022-10-31

**Authors:** Nathalie Dinganga Kapessa, Manix Ilunga Banza, Jeff Ntalaja, Kibangula Kasanga Trésor, Christelle Ngoie Ngoie, Daniel Bokar Nyamezawa, Tshilombo Katombe François, Willy Arung Kalau

**Affiliations:** 1 Université de Lubumbashi, Faculté de Médecine, Département de Chirurgie, Cliniques Universitaires de Lubumbashi, Lubumbashi, Haut Katanga, République Démocratique du Congo,; 2 Clinique Ngaliema, Kinshasa, République Démocratique du Congo

**Keywords:** Hydrocéphalie, dérivation ventriculopéritonéale, liquide céphalorachidien, Hydrocephalus, ventriculoperitoneal shunt, cerebrospinal fluid

## Abstract

**Introduction:**

l´hydrocéphalie est une distension progressive des espaces anatomiques (ventriculaires et sous arachnoïdiens) où siègent normalement le liquide céphalorachidien. Elle est plus fréquente chez les enfants. Dans les pays développés, sa prévalence et son incidence sont respectivement estimées entre 0,9 à 1,2 pour 1000 et 0,2 à 0,6 pour 1000 naissances vivantes et il y´aurait chaque année, entre 50 000 et 100 000 nouveaux cas d´hydrocéphalie dans le monde. L´objectif de cette étude était de décrire les aspects cliniques, paracliniques et thérapeutiques de l´hydrocéphalie dans 4 formations médicales à Lubumbashi.

**Méthodes:**

il s´est agi d´une étude descriptive transversale aux Cliniques Universitaires de Lubumbashi, à l´Hôpital du Cinquantenaire, à l´Hôpital Général de Référence Sendwe et à ArS Clinic, du 1^er^ avril 2015 au 30 septembre 2019. Les données ont été recueillies sur base d´une fiche de récolte des données ayant plusieurs paramètres d´études dont l´âge, le sexe, les signes cliniques, poids de naissance, antécédent, périmètre crânien, le bilan du scanner, l´évolution. Notre échantillon était de 91 cas d´hydrocéphalie.

**Résultats:**

la tranche d´âge de [29 jours à 24 mois] (nourrisson) était la plus fréquente soit 57,14% avec un sex ratio de 1,67 à prédominance masculine. Le principal signe de découverte était la macrocrânie chez tous nos patients suivis du regard en coucher de soleil chez 53,85% des patients. La tomodensitométrie cérébrale a été réalisée chez tous les patients et 65,92% présentaient l´hydrocéphalie tétraventriculaire. La dérivation ventriculopéritonéale a été réalisée chez tous les patients. L´évolution post-opératoire n´était émaillée d´aucun décès ; les complications post opératoires infectieuses et mécaniques ont représenté respectivement 8,79% et 4,40%. La durée moyenne d´hospitalisation était de 5,65 jours.

**Conclusion:**

l´hydrocéphalie reste la pathologie majeure de la neurochirurgie pédiatrique. Ces résultats doivent susciter l´attention des cliniciens pour un diagnostic précoce et une prise en charge correcte.

## Abstract (en)

**Introduction:**

hydrocephalus is a progressive distension of the anatomical spaces (ventricles and subarachnoid space) containing the cerebrospinal fluid. It most commonly affects children. In developed countries, its prevalence and incidence are estimated between 0.9 to 1.2 per 1000 and 0.2 to 0.6 per 1000 live births respectively and between 50 000 and 100 000 new cases develop each year in the world. The purpose of this study was to describe the clinical, paraclinical and therapeutic features of hydrocephalus in 4 medical facilities in Lubumbashi.

**Method:**

we conducted a descriptive cross-sectional study at the University Clinics of Lubumbashi, Cinquantenaire Hospital, Sendwe General Reference Hospital and ARS Clinic from April 1^st^, 2015 to September 30^th^, 2019. Data were collected on the basis of a data collection sheet containing several study parameters including age, sex, clinical signs, birth weight, patient’s history, head circumference, CT scan assessment and disease progression. Our sample consisted of 91 subjects with hydrocephalus

**Result:**

the age group 29 days to 24 months (infant) was most commonly affected, i.e. 57.14%, with a sex ratio of 1.67 and a male predominance. The main detecting sign was macrocrania in all patients, followed by setting-sun sign in 53.85% of patients. Brain CT scan was performed in all patients and 65.92% of them had tetraventricular hydrocephalus. Ventriculoperitoneal shunt was performed in all patients. There were no deaths in the postoperative period; postoperative infectious and mechanical complications accounted for 8.79% and 4.40% respectively. The average length of stay in the hospital was 5.65 days.

**Conclusion:**

hydrocephalus is the most common reason for pediatric neurosurgery. Clinicians should focus on these results which highlight the importance of early diagnosis and proper management.

## Introduction

L´hydrocéphalie est une ventriculomégalie à pression intracrânienne élevée par soit un excès de production, soit un défaut de réabsorption ou encore un obstacle à la circulation normale du liquide céphalo-rachidien, à début anté ou post natal, de type communicant ou non communicant et susceptible de comprimer le cerveau [1].

La fréquence de l´hydrocéphalie varie d´un pays à un autre. Dans les pays développés, sa prévalence et son incidence sont respectivement estimées entre 0,9 à 1,2 et 0,2 à 0,6 pour 1000 naissances vivantes. Il y´aurait chaque année, entre 50 000 et 100 000 nouveaux cas d´hydrocéphalie dans le monde [2]. Dans les pays développés, l´incidence de l´hydrocéphalie congénitale est de 0,5 à 1 /1000 naissances vivantes tandis que celle de l´hydrocéphalie acquise néonatale est de 3-5 /1000 naissances vivantes [3,4]. 6000 nouveaux cas par an en Afrique orientale, 45000 nouveaux cas par an d´Hydrocéphalie pédiatrique dans l´Afrique subsaharienne et 2000 à 4000 nouveaux cas pédiatriques par an en Ethiopie [5]. En République Démocratique du Congo, Tutukyona a réalisé une étude dans l´unité de neurochirurgie des Cliniques Universitaires de Kinshasa du 1^er^ juin 1990 au 30 juin 2013; il a trouvé une prévalence de 17,3% et une moyenne annuelle de 7,7 cas [6]. Les études récentes sur l´hydrocéphalie de l´enfant estiment l´incidence de 1,1 pour 1000 naissances vivantes dans le monde [7]. Néanmoins, il faudra noter qu´il est difficile de trouver l´incidence exacte de l´Hydrocéphalie, certaines études affirmant même que l´incidence actuelle en Afrique subsaharienne est inconnue [3].

L´objectif de notre étude était d´étudier l´hydrocéphalie de l´enfant dans notre milieu, depuis sa survenue, décrire les aspects cliniques et paracliniques, identifier les causes, sa prise en charge et analyser les suites post opératoires. Ainsi nous mettrons en évidence l´expérience de nos services en matière de la prise en charge des enfants hydrocéphales.

## Méthodes

Nous avons mené une étude descriptive transversale aux Cliniques Universitaires de Lubumbashi, à l´Hôpital Général de Référence Sendwe, à l´Hôpital du Cinquantenaire et à ArS Clinic du 1^er^ avril 2015 au 30 septembre 2019 sur 91 enfants. Les critères d´inclusion dans l´étude étaient les suivants: avoir moins de 18 ans et souffrant d´hydrocéphalie confirmée par le scanner cérébral; être opéré dans l´une des 4 institutions sanitaires ci-haut citées.

L´échantillonnage était exhaustif à l´Hôpital Provincial Général de Référence Sendwe et à l´Hôpital du Cinquantenaire où étaient réalisées deux campagnes gratuites pour prise en charge de l´hydrocéphalie du 1^er^ avril 2015 au 31 juillet 2017 dans le respect des critères d´inclusion sus mentionnés. Aux Cliniques Universitaires de Lubumbashi et à ArS Clinic du 1^er^ septembre 2017 au 30 septembre 2019, l´échantillonnage était aussi exhaustif suivant les mêmes critères d´inclusion sus mentionnés.

**Les variables de l´étude:** âge (jour, mois et année) et classes d´âge: répartition tenant compte de la croissance de l´enfant en nouveau-né (0 à 28 jours), nourrisson (29 jours à 2 ans), petit enfant de 2 à 5 ans, grand enfant de 6 à 11 ans et pubère et adolescent de 12 à 18 ans; sexe: la plupart des études montrent une prédominance du sexe masculin, cette prédominance s´explique en partie par le fait que l´hydrocéphalie congénitale peut se transmettre sur un mode récessif lié au sexe; le poids de naissance en gramme; les antécédents; périmètre crânien en centimètre (cm) mesuré par un mètre ruban au niveau de la plus grande circonférence. Selon l´Organisation Mondiale de la Santé (OMS), elle est de 34 cm plus ou moins 1 chez la fille et de 34,5 plus ou moins 1 chez le garçon à la naissance. La macrocrânie est définie par un périmètre crânien au-dessus de 2 déviations standards par rapport à la normale (10); le pourcentage d´augmentation du périmètre crânien par rapport à la moyenne du périmètre crânien normal suivant l´âge et le sexe défini par l´OMS; bombement des fontanelles; dilatation des veines épicrâniennes; les signes neurologiques et oculaires; les résultats de scanner cérébral; la prise en charge a 2 volets:

**Traitement médical:** une antibioprophylaxie faite du cefotaxime à raison de 100 milligrammes par kilogramme de poids, 3 fois par jour pendant 48 heures et à partir du premier coup de bistouri où le patient recevait le double de la dose prescrite. Un antalgique (paracétamol suppositoire à raison de 20mg par kilogramme de poids 2 fois par jour) en post-opératoire.

Traitement chirurgical était réalisé par La dérivation ventriculopéritonéale (DVP) par le shunt Chhabra (Chhabra®, Medipro Médical, Inde) Le shunt de Chhabra est un dispositif de drainage développé et fabriqué en Inde, qui intègre un système de dérivation d´hydrocéphalie à fente et à ressort complet, de taille standard avec un siphon gravitationnel empêchant un mécanisme effet siphon (certaines valves entrainent l´effet siphon c´est-à-dire un hyper drainage quand on passe de la position couchée à la position assise ou debout). Il est moins cher et est utilisé dans plus de 50 pays dans le monde, généralement dans les pays en voie de développement.

Un Chhabra “fente en ressort” est constitué de: une valve avec grand réservoir de rinçage; un drain péritonéal de 75 cm de long radio-opaque; un drain ventriculaire de 15 cm de long radio-opaque également; trois connecteurs droits.

Emballé et stérilisé dans des plateaux blister ouverts à double peau. Les patients bénéficiant de la DVP étaient préparés comme suit: cheveux coupés sur le site opératoire la veille de l´intervention chirurgicale; nettoyage des sites chirurgicaux (tête, cou, thorax et abdomen) par du savon Dettol, suivi du Dermobacter et le badigeonnage à la Bétadine au bloc opératoire; peau environnante drapée exposant uniquement les sites chirurgicaux; double gantage; shunt Chhabra utilisé chez tous nos patients avec comme précaution de changer de gants avant son utilisation; vérification de la fonctionnalité du shunt avant son insertion; libre circulation du LCR vérifiée avant fixation du cathéter ventriculaire avec le tube de dérivation.

L´évolution à court terme (durant les quatorze premiers jours post opératoires): durée d´hospitalisation, complications post opératoires précoces (infectieuses et mécaniques) et le traitement post-opératoire

## Résultats

### Données épidémio-cliniques

***Sexe et âge:*** le sexe masculin s´est révélé le plus fréquemment concerné par l´hydrocéphalie (62,64%). Le sexe ratio étant de 1,67 en faveur des garçons. En ce qui concerne l´âge: La tranche d´âge comprise entre [29 jours et 2 ans] (Nourrissons) a constitué le groupe d´âge le plus représenté avec 52 cas (soit 57,14%), suivi de celle allant de [2 à 5 ans] (petit enfant) avec 19 cas (soit 20,88%). La moyenne d´âge des patients de cette étude était de 2,45 ans (extrêmes allant de 3 jours à 16 ans). L´âge médian était de 1 an (premier quartile 13,5 mois et troisième quartile 4,5 ans) Tableau 1.

**Tableau 1:** répartition de l’âge en tranche

Tranche d’âge	Fréquence	%
Nouveau-né [0-28 jours]	6	6,59
Nourrisson [29 jours-2 ans]	52	57,14
Petit enfant [2-5 ans]	19	20,88
Grand enfant [6-12 ans]	12	13,19
Pubère et adolescent [13-18 ans]	2	2,20
**Total**	**91**	**100,00**

Le tableau ci-dessus montre un pic dans la tranche d’âge des nourrissons soit 57,14%. La moyenne d’âge des patients de cette étude était de 2,45 ans (extrêmes allant de 3 jours à 16 ans). L’âge médian était de 1 an (premier quartile 13,5 mois et troisième quartile 4,5ans).

***Poids de naissance, antécédent:*** vingt-deux patients soit 24,18% présentaient un poids inférieur à 2500 grammes. La méningite a été diagnostiquée comme antécédent dans 3 cas soit 3,30%. Il y a 8 mères qui ont suivi les CPN durant les 3 trimestres de grossesse; sept mères reconnaissent avoir pris l´acide folique pendant la grossesse mais cependant aucune de 7 n´en a pris au cours du premier trimestre. Il faut noter que 5,49% des mères ont souffert des infections génitales pendant la grossesse. Aucune notion de consanguinité n´a été notée chez les parents de nos patients.

***Signes cliniques:*** périmètre crânien: tous les patients avaient un périmètre crânien augmentés; pourcentage d´augmentation du périmètre crânien: cinquante patients soit 54,95% avaient une augmentation du périmètre crânien inférieur à 25% (Tableau 2); fontanelle antérieure bombée: Septante et un patients soit 78,02% avaient une fontanelle antérieure bombée; dilatations des veines épicrâniennes: cinquante-huit pourcent de nos patients présentaient une dilatation des veines épicrâniennes; signes neurologiques: vingt-sept patients soit 29,67% présentaient une difficulté de marcher alors que 22 d´entre-eux (24,17%) ne présentaient aucun signe neurologique (Tableau 3); signes oculaires: quarante-neuf enfants soit 53,85% présentaient un regard en coucher de soleil.

**Tableau 2:** pourcentage d’augmentation du périmètre crânien

Pourcentage d’augmentation du PC	Fréquence	%
<25%	50	54,95
25-50%	32	35,16
>50%	9	9,89
**Total**	**91**	**100,00**

Cinquante patients soient 54,95% avaient une augmentation du périmètre crânien inférieur à 25%

**Tableau 3:** signes neurologiques dominants

Signes neurologiques	Fréquence	%
Hypertonicité des membres inférieurs	9	9,89
Hypotonicité des membres inférieurs	12	13,18
Manque de tonicité axiale	10	10,99
M3 aux membres inférieurs	10	10,99
M4 aux membres inférieurs	1	1,09
Marche impossible	27	29,67
Aucun	22	24,17
**Total**	**91**	**100,00**

Vingt-sept patients soit 29,67 % présentaient une difficulté de marcher

### Données paracliniques

Tous nos patients avaient réalisé le scanner cérébral. Ces résultats nous ont permis de confirmer le diagnostic clinique et d´en établir l´étiologie pour certains. La majorité des patients soit 65,92% présentait une Hydrocéphalie tétraventriculaire Tableau 4.

**Tableau 4:** type d’hydrocéphalie

Type d’hydrocéphalie	Effectif	Pourcentage (%)
Biventriculaire	9	9,89
triventiculaire	22	24,17
tétraventriculaire	60	65,92
**Total**	**91**	**100**

L’hydrocéphalie tétra ventriculaire est la plus fréquente avec 65,92% des cas

### Données thérapeutiques et évolution

***Prise en charge:*** tous nos patients avaient subi une dérivation ventriculopéritonéale. Quatre patients ont été réopérés après déconnexion du drain. Tous nos patients avaient bénéficié d´une antibioprophylaxie et antalgique tel que présenté dans la méthodologie; complications post opératoires: huit virgule septante neuf pourcent des patients présentaient une infection des plaies opératoires; durée d´hospitalisation: la majorité des patients soit 85,71% ont séjourné 5 jours à l´hôpital; évolution à court terme tous les patients (100%) ont eu une bonne évolution et ont quitté l´hôpital. Aucun décès n´a été enregistré dans notre série.

## Discussion

Généralement, l´incidence de l´hydrocéphalie est identique dans les deux sexes sauf dans le syndrome de Bicker-Adam qui est transmis de façon mendélienne récessive lié au chromosome X. Ce gène transmis par le sexe féminin se manifeste seulement dans le sexe masculin [8]. Dans notre étude, la prédominance masculine de 62,64% avec un sex ratio de 1,67 comme dans l´ étude de Tapsoba avec un sex ratio de 1,52 à prédominance masculine [9] alors que d´autres études ont trouvé une prédominance féminine [3]. Dans notre étude, la moyenne d´âge est de 2,45 ans et 1 an d´âge médian alors que dans l´étude de Salem en Mauritanie, l´âge moyen au diagnostic est de 5 mois [3] et l´étude de Tapsoba où l´âge moyen est de 8,75 mois [9] beaucoup plus inférieur au notre.

Dans certains cas, le diagnostic étiologique de la méningite peut être perturbé si celle-ci est décapitée par un traitement antibiotique pour traiter une infection intercurrente. Ces hydrocéphalies peuvent être contemporaines de la phase aiguë ou survenir après une méningite apparemment guérie, surtout chez le nourrisson par blocage des espaces sous-arachnoïdiens et des citernes de la base. Dans notre étude 3 enfants soit 3,30% ont souffert de méningite avant la survenue de l´hydrocéphalie dont les germes n´ont pas été identifiés. Dans une étude réalisée en Côte d´Ivoire [10], l´hydrocéphalie représentant 54% des complications neurochirurgicales des méningites purulentes.

L´augmentation des apports en acide folique à un taux de 0.4 mg par jour en période périconceptionnelle entraine une diminution de moitié de l´incidence des anomalies de fermeture du tube neural. Cette supplémentation doit commencer 28 jours avant la conception et se poursuivre jusqu´à 12 semaines de gestation à la dose de 400 microgrammes par jour, il est recommandé de prescrire l´acide folique dès la connaissance de celle-ci. Dans notre étude, 8 femmes soit 8,79% ont suivi les CPN et 7 femmes 7,67% reconnaissent avoir pris l´acide folique pendant la grossesse mais aucune de 7 n´en a pris au cours du premier trimestre de grossesse.

Le rôle de la consanguinité dans la survenue de l´hydrocéphalie notamment congénitale n´est plus à démontrer [3]. Les signes cliniques de l´hydrocéphalie sont influencés par l´âge du patient, l´étiologie de l´hydrocéphalie, la localisation de l´obstruction et la durée d´évolution [11]. L´augmentation du périmètre crânien est le maitre symptôme de la pathologie chez l´enfant retrouvé par plusieurs auteurs. Cette augmentation est un mécanisme érigé par la voute crânienne de l´enfant, grâce à l´absence de fermeture de ses fontanelles, pour diminuer la pression intracrânienne. Elle peut être symétrique ou asymétrique (concerne la FCP en cas de malformation Dandy Walker - unilatérale en cas de la sténose d´un trou de Monro ou avec saillie frontale en cas de sténose de l´aqueduc de Sylvius) [12-14]. Elle peut être isolée ou accompagnée des perturbations neurologiques qu´il faut chercher par l´interrogatoire et l´examen clinique. Dans notre étude, tous nos patients présentaient une augmentation du périmètre crânien dont 54,95% une augmentation inférieure à 25% et 9,89% une augmentation supérieure à 50%.

L´augmentation du périmètre crânien reste le symptôme majeur de l´hydrocéphalie. Il existe inconstamment des signes discrets d´hypertension intracrânienne à type de tension de la fontanelle, de rétraction de la paupière supérieure. Ainsi dans notre série, 71 patients soit 78,02% avaient une fontanelle antérieure bombée et 58% présentaient une dilatation des veines épicrâniennes. Notre étude se rapproche des résultats de plusieurs études menées dans le monde [3,12,15]. L´enfant est souvent en pleurs et tonus axial, au début diminué, se trouve rapidement renforcé avec une hyper extension des membres inférieurs. Les troubles du comportement sont secondaires à la souffrance cérébrale qui est responsable du retard psychomoteur chez le nouveau-né et le nourrisson [3]. En ce qui concerne les signes d´HTIC, le petit enfant peut compenser une augmentation du volume intracrânien sans augmentation de la pression intracrânienne car les fontanelles sont ouvertes et les sutures non soudées, ce qui permet l´accroissement du PC. En revanche, quand l´augmentation de la pression intracrânienne survient, elle est beaucoup plus brutale que chez l´adulte car les espaces de résorption du LCR sont de faible volume. Les signes neurologiques sont dominés par la régression des acquisitions psychomotrices (perte de la station assise, la tenue de la tête et la station debout) chez la moitié des malades [16].

Dans notre série, les signes neurologiques étaient dominés par la difficulté de marcher chez 27 patients soit 29,67% et se rapprochent des études de Sidi Salem en Mauritanie où les troubles psychomoteurs représentaient 35,7% [3]. Le regard en coucher de soleil est un signe très fréquent, les globes oculaires basculent vers le bas sous l´effet de l´hyperpression qui s´exerce sur le toit des orbites, mais aussi sous l´effet de la paralysie de la verticalité du regard ou syndrome de Parinaud, ce qui permet de voir largement la cornée au-dessus de l´iris en partie masquée par la paupière inférieure [3,17]. Le strabisme convergeant souvent observé, est rapporté à une atteinte du sixième nerf crânien par hypertension. La baisse de l´acuité visuelle est difficile à dépister chez les nourrissons, seule l´absence de la poursuite oculaire ou la présence de mouvements désordonnés des yeux sont évocateurs d´une amblyopie grave qui peut être la conséquence d´une atrophie optique secondaire à l´hydrocéphalie évoluée de diagnostic trop tardif [17]. Dans notre étude, 49 enfants soit 53,85% présentaient un regard en coucher de soleil alors que Salem lui trouve 35,7% d´enfant avec regard en coucher de soleil [3].

La tomodensitométrie est l´examen fondamental qui permet d´affirmer la dilatation des ventricules, de la mesurer avec précision, d´apprécier son caractère global ou segmentaire, bi- ou triventriculaire, orientant ainsi la recherche étiologique et la demande d´autres investigations. Le risque d´irradiation est modeste mais ne doit pas être négligé: la toxicité pour le cristallin, par exemple, apparaît à partir du quinzième examen. L´examen sans injection est le plus souvent suffisant, mais l´injection intraveineuse de produit iodé est parfois indispensable pour rechercher une malformation vasculaire ou une tumeur, en préciser la localisation par rapport aux structures méningées et vasculaires. Aux coupes obliques selon le plan orbitoméatal, ou horizontales, parallèles à l´axe des voies optiques, il peut être utile d´adjoindre des coupes coronales, vertico-frontales, pour l´étude de lésions profondes sus-tensorielles, et parfois sagittales, utiles dans les lésions de la fosse postérieure et de la ligne médiane [18-21]. Dans notre étude la TDM cérébrale été réalisé chez 91 patients soit 100%.

L´hydrocéphalie tétraventriculaire était présente chez 29,67% de nos patients et suivi de la sténose de l´aqueduc de Sylvius chez 18,68 % et la maladie de Dandy-walker a représenté 7,69% de nos patients par contre dans l´étude de Sadi Salem la sténose de l´aqueduc de Sylvius a représenté 11,9% et la maladie de Dandy Walker a représenté 13,4% [3] . La DVP est le traitement habituel de l´hydrocéphalie [22,23]. Cette modalité thérapeutique a largement acquis ses lettres de noblesse mais n´a pas résisté à l´expansion de la ventriculocisternostomie endoscopique (VCE) dans les indications spécifiques telles que la sténose de l´aqueduc de Sylvius. Son but est de drainer le LCS depuis le 3^e^ventricule vers les citernes de la base du crâne. Elle est largement utilisée chez l´adulte et l´enfant. Son avantage est d´éviter les risques infectieux et mécaniques liés à l´implantation d´une prothèse [24]. Elle est encore limitée et onéreuse dans certains pays africains [23] et indisponible dans notre pays. Le traitement idéal est celui de la cause quand il est accessible: exérèse d´une tumeur, traitement d´une malformation vasculaire, levée d´un obstacle au retour veineux. L´hydrocéphalie installée peut évoluer pour son propre compte, même après traitement de la cause. C´est pourquoi un geste de dérivation est habituellement nécessaire [15,25].

Dans notre étude, tous les patients avaient subi une dérivation ventriculopéritonéale et une antibioprophylaxie faite du cefotaxime. Quatre patients ont été réopérés soit 4,39 % après déconnexion du drain. La DVP était également la seule technique chirurgicale utilisée dans l´étude menée par Tutukyona à Kinshasa [6]. Dans la série de Laeke en Ethiopie, 74,7% des patients ont subi une DVP et 23,3% ont subi VCE, une antibioprophylaxie faite de ceftriaxone et 52,4% ont été réopéré pour échec du shunt [5]. Dans les études de Warf en Ouganda en 2005 et beaucoup d´autres, la VCE avait été aussi réalisée [14,23,26]. La VCE était préférée chez le nourrisson dans l´étude de Salem (74,1%) alors que la DVP elle était préférée chez le nouveau-né [3]. Dans notre étude, 8,79% des patients ont présenté une infection au site opératoire et 4,40% de complication mécanique.

Nos résultats paraissent bons comparés à ceux de: Tutukyona à Kinshasa avec un taux des complications infectieuses de 18,8% et complication mécanique à 19,80% [6]; Mouafo au Cameroun avec 22,8% de complication infectieuse [22]; Topzeweska en Pologne avec 15,20% de complication infectieuse contre 36,9% de complications mécaniques [10]; Salem en Mauritanie a trouvé un taux global des complications de 26,1% [3];

Ces différences peuvent être liées aux précautions per opératoire utilisées, au délai de suivi des patients et aux types de valves utilisées. L´hydrocéphalie reste ainsi donc une pathologie fréquente en neurochirurgie pédiatrique et représente un facteur important de morbi-mortalité [27]; mais la DVP reste le traitement de choix des hydrocéphalies [3]. Dans l´étude de Paulsen, sur une longue période de suivi de 42-45 ans, le taux de mortalité était de 48% [14]. Dans notre étude, la durée d´hospitalisation était de 5 jours pour 85,71% de nos patients avec une durée moyenne d´hospitalisation de 5,65 jours. Dans l´étude de Baykan en Turquie, la durée moyenne d´hospitalisation était de 3,15 jours [28]. Le délai d´hospitalisation se rapproche et serait dû à la bonne évolution des patients et à la prise en charge des patients par des mutuelles. Dans notre étude, 86,81% de nos patients ont eu une bonne évolution et 75,82% de nos patients ont été pris en charge lors des campagnes chirurgicales, de soins gratuits. La mortalité postopératoire (DVP) était nulle dans notre étude. Elle a été constatée par Tabaski [11] en Tunisie 20,3%, par Christina aux Etats Unis 1,6%, par Warf en Ouganda 15,9%. Avec la VCE, Warf a trouvé une mortalité faible de 1,8% [29]. Ces différences peuvent être dues au suivi des malades au long cours. Dans notre étude l´évolution était à court terme, limitée au dernier pansement du patient, avant sa sortie de l´hôpital.

Dans notre étude, 78 patients soit 85,71% ont séjourné 5 jours à l´hôpital en post opératoire sans complication précoce. Dans les séries de l´Afrique subsaharienne, les complications décrites varient entre 7% et 69% et sont liées à des causes mécaniques (11-54%) et à l´infection (7-69%) [3]. Plusieurs études parlent de réopération après échec du shunt de dérivation [30] et ces échecs conduisent à la réopération du patient [31] et prolonge le séjour du patient. Les complications mécaniques (déconnexion, migration du drain peritoneal…) et les infections (méningites, péritonites,…) nécessitent une réopération du patient et prolonge son séjour à l´hôpital.

Un travail méticuleux en salle d´opération élimine l´infection par shunt: technique chirurgicale uniforme avec manipulation limitée de l´implant et de la peau; circulation réduite en salle d´opération; planification matinale avec une durée d´intervention de moins de 30 minutes; double gantage, retirer la paire de gants externe avant de manipuler le shunt; antibioprophylaxie systématique.

## Conclusion

L´hydrocéphalie est une affection redoutable qui se répercute sur le développement psychomoteur et les capacités intellectuels de l´enfant, l´empêchant ainsi d´intégrer normalement la société. Dans notre étude, le sexe masculin était dominant avec 62,64% de patients. Les nourrissons étaient la tranche d´âge la plus touchée soit 57,14, et 53,85% de patients avaient un regard en coucher de soleil et 3,30% de malformations associées. Tous nos patients avaient réalisé le scanner cérébral avec 65,92% d´hydrocéphalie tétraventriculaire suivi de 24,68% d´hydrocéphalie triventriculaire par sténose de l´aqueduc de Sylvius. Tous nos patients avaient bénéficié d´une dérivation ventriculopéritonéale. Les complications infectieuses post opératoires étaient de 8,79% et les complications mécaniques de 4,40% avec une durée moyenne d´hospitalisation de 5,65 jours. Les patients sans complications ont séjourné moins de jours par rapport à ceux avec complications.

### Etat des connaissances sur le sujet

- L´hydrocéphalie reconnaît une multitude d´étiologies, le plus souvent malformative et hémorragique en période néonatale, post-méningitique chez le nourrisson et tumorale chez le grand enfant;
- Le scanner cérébral et l´imagerie par résonnance magnétique) contribuent suffisamment au diagnostic;
- La dérivation ventriculopéritonéale est la technique la plus utilisée en Afrique subsaharienne.

### Contribution de notre étude à la connaissance

- Il s´agit de la première étude sur l´hydrocéphalie de l´enfant avec plus de 50 cas à Lubumbashi (2^e^ ville de la République Démocratique Du Congo).

## Remerciements

Nous remercions Madame Olive Lembe (ex première dame de la République Démocratique du Congo) pour avoir pris en charge les consultations, le scanner cérébral, la prise en charge chirurgicale et l´hospitalisation de 75,82% de nos patients.

## References

[1] Sainte-Rose C, Zerah M, Puget S, Di Rocco F, Blauwblomme T, De Olivera R (2012). Vingt ans de traitement de l´hydrocéphalie chez l´enfant. Neurochirurgie.

[2] Garton HJL, Piatt JH (2004). Hydrocephalus. Pediatr Clin North Am.

[3] Salem-Memou S, Chavey S, Elmoustapha H, Mamoune A, Moctar A, Salihy S (2020). [Hydrocephalus in newborns and infants at the Nouakchott National Hospital]. Pan Afr Med J.

[4] Chi JH, Fullerton HJ, Gupta N (2005). Time trends and demographics of deaths from congenital hydrocephalus in children in the United States: National Center for Health Statistics data, 1979 to 1998. J Neurosurg.

[5] Laeke T, Tirsit A, Biluts H, Murali D, Wester K (2017). Pediatric Hydrocephalus in Ethiopia: Treatment Failures and Infections: A Hospital-Based, Retrospective Study. World Neurosurg.

[6] Tutukyona IJ, Kalubie BA, Ntombo BJ, Glennie EN (2018). Aspects épidémiologiques, cliniques et thérapeutiques de l´hydrocéphalie aux Cliniques Universitaires de Kinshasa. Ann Afr Médecine.

[7] Tully HM, Dobyns WB (2014). Infantile hydrocephalus: a review of epidemiology, classification and causes. Eur J Med Genet.

[8] Gathura E, Poenaru D, Bransford R, Albright AL (2010). Outcomes of ventriculoperitoneal shunt insertion in Sub-Saharan Africa. J Neurosurg Pediatr.

[9] Tapsoba TL, Sanon H, Soubeiga KJ, Ouattara TF, Kabré A, Cissé R (2010). Aspects épidémiologiques, cliniques et tomodensitométriques des hydrocéphalies chez les enfants de zéro à 15 ans (à propos de 53 patients colligés au centre hospitalier universitaire Yalgado Ouédraogo de Ouagadougou: CHU YO). Médecine Nucl.

[10] Moutard M-L, Gelot A (2006). Diagnostic prenatal d´anomalies cérébrales de pronostic incertain, que dire-?. que faire? Arch Pédiatrie.

[11] Tabaski B (2001). Hydrocéphalie de L´Enfant, aspects étiologiques et évolutifs: à propos de 86 observations. Rev Maghrebine Pédiatrie.

[12] McLone DG (2000). Hydrocephalus. Pediatr Neurosurg.

[13] Wright Z, Larrew TW, Eskandari R (2016). Pediatric Hydrocephalus: Current State of Diagnosis and Treatment. Pediatr Rev.

[14] Paulsen AH, Lundar T, Lindegaard K-F (2015). Pediatric hydrocephalus: 40-year outcomes in 128 hydrocephalic patients treated with shunts during childhood. Assessment of surgical outcome, work participation, and health-related quality of life. J Neurosurg Pediatr.

[15] Yamasaki M, Nonaka M (2009). [Diagnosis and treatment of congenital hydrocephalus]. No Shinkei Geka.

[16] Bergsneider M, Egnor MR, Johnston M, Kranz D, Madsen JR, Mcallister JP (2006). What we don´t (but should) know about hydrocephalus. J Neurosurg Pediatr.

[17] Swash M (1976). Disorders of ocular movement in hydrocephalus. Proc R Soc Med.

[18] Schwalbe J, Hofmann V (1986). [Possibilities of pre-, intra-and postoperative sonography in hydrocephalus]. Z Kinderchir Organ Dtsch Schweiz Osterreichischen Ges Kinderchir Surg Infancy Child.

[19] Dinçer A, Özek MM (2011). Radiologic evaluation of pediatric hydrocephalus. Childs Nerv Syst ChNS Off J Int Soc Pediatr Neurosurg.

[20] O´Neill BR, Pruthi S, Bains H, Robison R, Weir K, Ojemann J (2013). Rapid Sequence Magnetic Resonance Imaging in the Assessment of Children with Hydrocephalus. World Neurosurg.

[21] Ashley WW, McKinstry RC, Leonard JR, Smyth MD, Lee BC, Park TS (2005). Use of rapid-sequence magnetic resonance imaging for evaluation of hydrocephalus in children. J Neurosurg.

[22] Tambo FFM, Djientcheu V, Chiabi A, Mbarnjuk SA, Walburga YJ, Mbonda E (2011). Our experience in the management of infantile hydrocephalus: a study on thirty-five regrouped cases in Yaounde, Cameroon. Afr J Paediatr Surg AJPS.

[23] Warf BC, East African Neurosurgical Research Collaboration (2010). Pediatric hydrocephalus in East Africa: prevalence, causes, treatments, and strategies for the future. World Neurosurg.

[24] El-Ghandour NMF (2011). Endoscopic third ventriculostomy versus ventriculoperitoneal shunt in the treatment of obstructive hydrocephalus due to posterior fossa tumors in children. Childs Nerv Syst ChNS Off J Int Soc Pediatr Neurosurg.

[25] Garegnani L, Franco JV, Ciapponi A, Garrote V, Vietto V, Portillo Medina SA (2020). Ventriculo-peritoneal shunting devices for hydrocephalus. Cochrane Database Syst Rev 16.

[26] Chiafery M (2006). Care and management of the child with shunted hydrocephalus. Pediatr Nurs.

[27] Muir RT, Wang S, Warf BC (2016). Global surgery for pediatric hydrocephalus in the developing world: a review of the history, challenges, and future directions. Neurosurg Focus.

[28] Baykan N, Isbir O, Gerçek A, Dagçnar A, Ozek MM (2005). Ten years of experience with pediatric neuroendoscopic third ventriculostomy: features and perioperative complications of 210 cases. J Neurosurg Anesthesiol.

[29] Warf BC, Kulkarni AV (2010). Intraoperative assessment of cerebral aqueduct patency and cisternal scarring: impact on success of endoscopic third ventriculostomy in 403 African children. J Neurosurg Pediatr.

[30] Enger PØ, Svendsen F, Sommerfelt K, Wester K (2005). Shunt revisions in children--can they be avoided-Experiences from a population-based study. Pediatr Neurosurg.

[31] Komolafe EO, Adeolu AA, Komolafe MA (2008). Treatment of cerebrospinal fluid shunting complications in a Nigerian neurosurgery programme, Case illustrations and review. Pediatr Neurosurg.

